# Prevention of the first episode of psychosis: What have we reached by 2021?

**DOI:** 10.1192/j.eurpsy.2021.221

**Published:** 2021-08-13

**Authors:** M. Rojnic-Kuzman

**Affiliations:** Department Of Psychiatry, Zagreb School of Medicine and ZagUniversity Hospital Centre, Zagreb, Croatia

## Abstract

**Abstract Body:**

Prevention of the First Episode of Psychosis: What Have we Reached by 2021? The first episode of psychosis is usually preceded by a prodromal period or stage of psychosis, where early signs of symptoms indicating onset of first episode psychosis (FEP) occur. In the last twenty years, enormous progress was made in the tretment of FEP and subsequently schizophrenia, as the focus of treatment of FEP shifted to this prodromal period with the aim of preventing the first episode of psychosis in people at risk.Treatment for the prodromal stage of psychosis is provided within specialized early intervention services, which are somtimes part of the services for the treatment of FEP. Early intervention services, which have been gradually developed in many countries worldwide, usually incorporate multimodal treatment approaches (pharmacotherapy, psychotherapy and psychosocial interventions). However, there are still many differences in the treatment of prodromes across early intervention services, even within one country, leaving open the questions on what kind or combinations of treatments really work in the prevention of FEP. The methods of studies in the scientific psychiatric literature do not allow easy translation of scientific data to clinical practice. In the presentation, an up-to-date overview of the available treatments offered witin early intervention services for prevention of FEP is given.

**Disclosure:**

No significant relationships.

